# Supplementary material for: Evaluation of the reduction in perceived and performance fatigability by functional compression tights during squat exercises via electromyography and electroencephalography analysis
Source: PLoS One. 2025 Sep 5;20(9):e0331778. doi: 10.1371/journal.pone.0331778 (PMC12412955; doi:10.1371/journal.pone.0331778)
Supplement: Attachment — Submitted filename: Response_to_Reviewers_auresp_2.docx [file pone.0331778.s005.docx]

29-July-2025

Dr. Emily Chenette

Editor-in-Chief

Dr. Hasan Sozen

Academic Editor

*PLOS ONE*

**Manuscript for submission: "Evaluation of the reduction in perceived and performance fatigability by functional compression tights during squat exercise via electromyography and electroencephalography analysis" (PONE-D-25-23745)**

Dear Editor,

We would like to express our sincere appreciation for the valuable comments and suggestions provided by the reviewers regarding our manuscript entitled:

“Evaluation of the reduction in perceived and performance fatigability by functional compression tights during squat exercise via electromyography and electroencephalography analysis” (PONE-D-25-23745).

In response to the reviewer comments, we have thoroughly revised the manuscript. Major modifications include:

- Clarification and standardization of terminology related to fatigue and fatigability throughout the manuscript, including the abstract, based on the taxonomy proposed by Enoka and Duchateau (2016).
- Replacement of the term “subjective fatigue” with the more accurate expression “perceived fatigability” where appropriate.
- Correction of p-value formatting, ensuring consistency to three decimal places across the abstract, results, tables, and figure legends.
- Removal of all instances of ambiguous statistical phrasing such as “trend toward significance,” in line with reviewer recommendations.
- Consistent use of the term “vastus medialis obliquus (VMO)” throughout the text.
- Complete revision of all figures to ensure English-only labeling and improved font sizes for readability. All revisions in the manuscript are highlighted in red. We have also updated the reference format to comply with the Vancouver style as per journal requirements.

All revisions are clearly marked using Track Changes in the manuscript.

We are grateful for the reviewers’ and editors’ comments, which have greatly improved the clarity and scientific rigor of our manuscript. We sincerely hope that the revised version is now suitable for publication in PLOS ONE.

Sincerely,

Hayato Shigetoh, PhD

Department of Physical Therapy

Faculty of Health Science

Kyoto Tachibana University

Yamashina-ku, Kyoto, Japan

Tel.: +81-75-571-1111

Email: [shigeto@tachibana-u.ac.jp](mailto:shigeto@tachibana-u.ac.jp)

**Reviewer #1:**

**Reviewer comments:**

All of my requested revisions were considered. The authors increased the number of references and edited and improved some paragraphs.

**Response:**

Thank you very much for your constructive feedback and thoughtful comments throughout the review process. We are grateful that all of your requested revisions were considered acceptable. Your suggestions have contributed significantly to improving the clarity, quality, and overall rigor of the manuscript. We believe that your comments have helped us develop a stronger and more impactful paper.

**Reviewer #2:**

**Reviewer comments:**

I would like to thank the authors for answering most of my questions and suggestions for improving their manuscript. There are still a few points that need to be fixed so that the manuscript can then be accepted for publication.

**Response:**

Thank you for reviewing our manuscript. Your comments were highly insightful and enabled us to greatly improve the quality of our manuscript. In the following pages are our point-by-point responses to each of the comments of the reviewers as well as your own comments. Revisions in the text are shown using Track Changes. Also, the paper has been edited and proofread by a professional native English speaking editing service.

**Reviewer comments:**

1. During my initial revision, I suggested that the authors update the definition of fatigue to that proposed by Duchateau and Enoka 10 years ago. However, the authors did not make corrections throughout the manuscript, so in the current form there is a lot of confusion around the term fatigue and fatigability, because both terms are used.

For example, why did the authors not change the term “subjective fatigue” to the more correct and accurate term “perceived fatigability”? For example, what is “subjective lower limb fatigue”?

I again suggest that the authors be consistent with the terminology throughout the manuscript, including the abstract!

**Response:**

Thank you for your valuable comment regarding the terminology of fatigue and fatigability. In response to your suggestion, we carefully reviewed the manuscript and revised the terminology throughout to ensure consistency and accuracy based on the definition proposed by Duchateau and Enoka. Specifically, we introduced the definition of fatigability in the Introduction and subsequently replaced terms such as “subjective fatigue” with “perceived fatigability” where appropriate, including in the Abstract, Methods, Results, Discussion, and Conclusion. We appreciate your guidance, which helped us improve the conceptual clarity of the manuscript.

**Reviewer comments:**

2. p-values

Once again, I strongly suggest that the authors use three decimal places in all results. For example, in Table 2, the decimal p-values are still limited to two. Please amend.

**Response:**

Thank you for pointing this out again. In accordance with your suggestion, we have revised all p-values throughout the manuscript—including those in the Abstract, main text, tables, and figure legends—to consistently report values to three decimal places. We carefully re-checked each occurrence to ensure uniformity and precision in statistical reporting. We appreciate your attention to detail, which has contributed to the improvement of the manuscript’s clarity and scientific rigor.

**Reviewer comments:**

3. The issue about “trend toward significance”, which I pointed out in the first revision phase, has not been completely resolved. Please modify at lines 350-352.

**Response:**

Thank you for your insightful comment. In response, we thoroughly reviewed the manuscript, including the Abstract, Results, and Discussion sections, and removed or rephrased all instances of the expression “trend toward significance” as appropriate. These changes were made to ensure clarity and consistency in statistical reporting, and to avoid potentially ambiguous interpretations. We appreciate your guidance in improving the quality of the manuscript.

**Reviewer comments:**

4. Muscle terminology

The authors changed the term “vastus medialis” to the more precise “vastus medialis obliquus”. However, this has not been done throughout the manuscript. Please amend accordingly.

**Response:**

Thank you for your valuable suggestion. In accordance with your comment, we have carefully reviewed the entire manuscript and consistently replaced the term “vastus medialis” with the more precise term “vastus medialis obliquus” or its abbreviation “VMO” where appropriate. This ensures anatomical accuracy and clarity throughout the manuscript.

**Reviewer comments:**

5. Figures

Many figures still contain Japanese terms. Please change them to English.

Furthermore, the figures appear too small to be observed correctly by readers. Please edit.

**Response:**

Thank you for your helpful comment. In response, we thoroughly reviewed all figures to ensure that no Japanese terms remain and confirmed that all labels and annotations are now fully presented in English. Additionally, we have adjusted the font sizes and overall layout of each figure to improve visibility and clarity, ensuring they can be easily interpreted by readers. All revised figures have been resubmitted accordingly.

**Reviewer comments:**

Minor comments

L138, L149, L150, etc “a muscle fatigue task” --> a fatiguing task.

**Response:**

I have revised it.

**Reviewer comments:**

L184 Time --> time

**Response:**

I have revised it.

**Reviewer comments:**

L281 please add a space between “the” and “condition”.

**Response:**

I have revised it.
